# 4-Amino-3,5-dimethyl-4*H*1-,2,4-triazole–water (2/3)

**DOI:** 10.1107/S1600536808028146

**Published:** 2008-09-06

**Authors:** Lin Cheng, Ya-Wen Zhang, Yan-Yan Sun, Gang Xu

**Affiliations:** a Department of Chemistry and Chemical Engineering, Southeast University, Nanjing, People’s Republic of China

## Abstract

The asymmetric unit of the title compound, 2C_4_H_8_N_4_·3H_2_O, contains two crystallographically independent 4-amino-3,5-dimethyl-1,2,4-triazole mol­ecules and three water mol­ecules. The structure exhibits N—H⋯O, O—H⋯N and O—H⋯O hydrogen bonds.

## Related literature

For related structures, see: Wang *et al.* (2006 ▶); Zachara *et al.* 2004 ▶). For related literature, see: Beckmann & Brooker (2003 ▶); Bentiss *et al.* (1999 ▶); Collin *et al.* (2003 ▶); Curtis (2004 ▶).
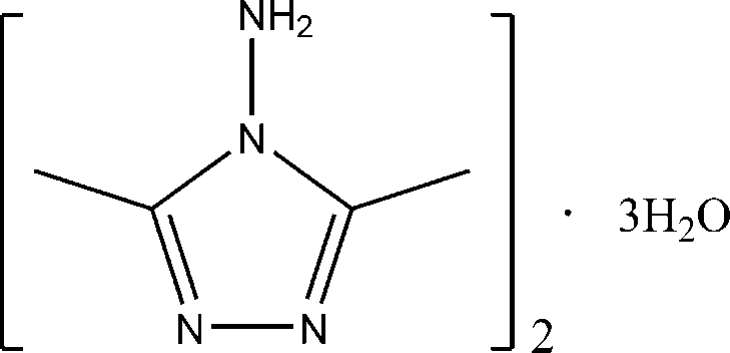


## Experimental

### 

#### Crystal data

2C_4_H_8_N_4_·3H_2_O
*M* *_r_* = 278.34
Triclinic, 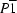

*a* = 7.194 (4) Å
*b* = 8.680 (4) Å
*c* = 13.592 (7) Å
α = 72.332 (8)°
β = 84.993 (8)°
γ = 68.936 (7)°
*V* = 754.5 (6) Å^3^
*Z* = 2
Mo *K*α radiation
μ = 0.10 mm^−1^
*T* = 293 (2) K
0.20 × 0.18 × 0.17 mm

#### Data collection

Bruker APEX CCD diffractometer
Absorption correction: multi-scan (*SADABS*; Sheldrick, 2000 ▶) *T*_min_ = 0.981, *T*_max_ = 0.984
5166 measured reflections
2904 independent reflections
2447 reflections with *I* > 2σ(*I*)
*R*_int_ = 0.014

#### Refinement

*R*[*F* ^2^ > 2σ(*F* ^2^)] = 0.049
*wR*(*F* ^2^) = 0.137
*S* = 1.03
2904 reflections
213 parameters
H atoms treated by a mixture of independent and constrained refinement
Δρ_max_ = 0.27 e Å^−3^
Δρ_min_ = −0.22 e Å^−3^

### 

Data collection: *SMART* (Bruker, 2000 ▶); cell refinement: *SAINT* (Bruker, 2000 ▶); data reduction: *SAINT*; program(s) used to solve structure: *SHELXS97* (Sheldrick, 2008 ▶); program(s) used to refine structure: *SHELXL97* (Sheldrick, 2008 ▶); molecular graphics: *SHELXTL* (Sheldrick, 2008 ▶); software used to prepare material for publication: *SHELXTL*.

## Supplementary Material

Crystal structure: contains datablocks I, global. DOI: 10.1107/S1600536808028146/bt2782sup1.cif

Structure factors: contains datablocks I. DOI: 10.1107/S1600536808028146/bt2782Isup2.hkl

Additional supplementary materials:  crystallographic information; 3D view; checkCIF report

## Figures and Tables

**Table 1:** Hydrogen-bond geometry (Å, °)

*D*—H⋯*A*	*D*—H	H⋯*A*	*D*⋯*A*	*D*—H⋯*A*
N4—H4*D*⋯O1*W*	0.93 (2)	2.00 (2)	2.924 (3)	170.9 (18)
N4—H4*E*⋯O3*W*^i^	0.88 (2)	2.21 (2)	3.078 (3)	168.3 (18)
N8—H8*E*⋯O2*W*^ii^	0.93 (3)	2.23 (3)	3.104 (3)	156 (2)
O1*W*—H1*WA*⋯O2*W*^iii^	0.84 (3)	1.95 (3)	2.793 (3)	173 (3)
O1*W*—H1*WB*⋯O3*W*^iv^	0.92 (3)	1.93 (3)	2.810 (2)	160 (3)
O2*W*—H2*WA*⋯N2	0.87 (3)	2.02 (3)	2.885 (2)	171 (2)
O2*W*—H2*WB*⋯N5	0.90 (3)	1.93 (3)	2.816 (2)	168 (2)
O3*W*—H3*WA*⋯N1	0.88 (2)	1.92 (2)	2.787 (2)	168 (2)
O3*W*—H3*WB*⋯N6	0.89 (3)	1.93 (3)	2.827 (2)	176 (2)

Symmetry codes: (i) 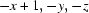; (ii) 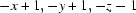; (iii) 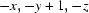; (iv) 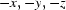.

**Fig. 1.**
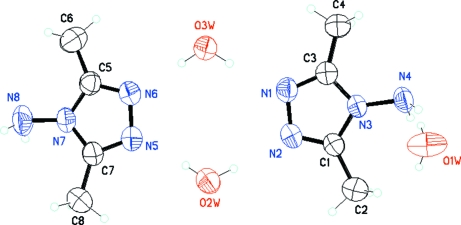
The asymmetric unit of the title compound with 30% thermal ellipsoids.

**Fig. 2.**
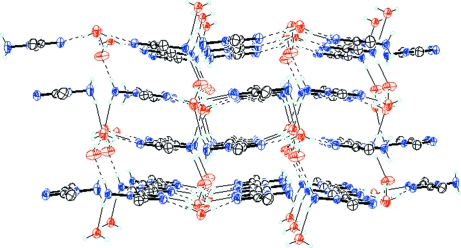
The three-dimensional supramolecular network of the title compound. The H atoms bonded to C atoms are omitted for clarity.

## supplementary crystallographic information

### Comment

The derivatives of 4-amino-1,2,4-triazoles have considerable importance in medicinal chemistry, agricultural and industrial chemistry (Bentiss *et al.* 1999; Collin *et al.* 2003; Curtis *et al.* 2004). They have also been used as multidentate ligands in coordination chemistry (Beckmann *et al.* 2003). Here, we report a hydrated 4-amino-1,2,4-triazole (mta)_2_.3H_2_O (mta = 4-amino-3,5-dimethyl-1,2,4-triazole).

The asymmetric unit of the title compound contains two crystallographically independent mta molecules and three water molecules. The C═N—N—C fragments of the tetrazine rings have the C═N distances of 1.299 (2), 1.300 (2) and 1.304 (2) Å, and the N—N distances of 1.392 (2) and 1.389 (2) Å. All other C—N distances are between 1.352 (2) and 1.362 (2) Å, which are considered to have part double-bond character. In the crystalline state, the mta and crystal water molecules are linked together by N—H···O, O—H···N and O—H···O hydrogen bonding.

### Experimental

To a solution of mta (mta = 4-amino-3,5-dimethyl-1,2,4-triazole) (0.0228 g, 0.2 mmol) in CH_3_OH (5 ml), an aqueous solution (5 ml) of MnSO_4_.H_2_O (0.0169 g, 0.1 mmol) was added. The mixture was stirred for half an hour and filtered. The filtrate was allowed to evaporate slowly at room temperature. After several days, colorless block crystals were obtained in 5% yield (0.0007 g) based on mta.

### Refinement

H atoms bonded to O and N atoms were located in a difference map and freely refined. Other H atoms were positioned geometrically and refined using a riding model with C—H = 0.96 Å and with *U*_iso_(H) = 1.5*U*_iso_(C).

### Crystal data

**Table:**

2C_4_H_8_N_4_·3H_2_O	*Z* = 2
*M**_r_* = 278.34	*F*(000) = 300
Triclinic, *P*1	*D*_x_ = 1.225 Mg m^−^^3^
Hall symbol: -P 1	Mo *K*α radiation, λ = 0.71073 Å
*a* = 7.194 (4) Å	Cell parameters from 785 reflections
*b* = 8.680 (4) Å	θ = 2.4–28.0°
*c* = 13.592 (7) Å	µ = 0.10 mm^−^^1^
α = 72.332 (8)°	*T* = 293 K
β = 84.993 (8)°	Block, colourless
γ = 68.936 (7)°	0.20 × 0.18 × 0.17 mm
*V* = 754.5 (6) Å^3^	

### Data collection

**Table:**

Bruker APEX CCD diffractometer	2904 independent reflections
Radiation source: fine-focus sealed tube	2447 reflections with *I* > 2σ(*I*)
graphite	*R*_int_ = 0.014
φ and ω scan	θ_max_ = 26.0°, θ_min_ = 2.6°
Absorption correction: multi-scan (SADABS; Sheldrick, 2000)	*h* = −8→8
*T*_min_ = 0.981, *T*_max_ = 0.984	*k* = −10→10
5166 measured reflections	*l* = −16→12

### Refinement

**Table:**

Refinement on *F*^2^	Secondary atom site location: difference Fourier map
Least-squares matrix: full	Hydrogen site location: inferred from neighbouring sites
*R*[*F*^2^ > 2σ(*F*^2^)] = 0.049	H atoms treated by a mixture of independent and constrained refinement
*wR*(*F*^2^) = 0.137	*w* = 1/[σ^2^(*F*_o_^2^) + (0.0782*P*)^2^ + 0.0952*P*] where *P* = (*F*_o_^2^ + 2*F*_c_^2^)/3
*S* = 1.03	(Δ/σ)_max_ < 0.001
2904 reflections	Δρ_max_ = 0.27 e Å^−^^3^
213 parameters	Δρ_min_ = −0.22 e Å^−^^3^
0 restraints	Extinction correction: SHELXL97 (Sheldrick, 2008), Fc^*^=kFc[1+0.001xFc^2^λ^3^/sin(2θ)]^-1/4^
Primary atom site location: structure-invariant direct methods	Extinction coefficient: 0.151 (12)

### Special details

**Table:**

Geometry. All e.s.d.'s (except the e.s.d. in the dihedral angle between two l.s. planes) are estimated using the full covariance matrix. The cell e.s.d.'s are taken into account individually in the estimation of e.s.d.'s in distances, angles and torsion angles; correlations between e.s.d.'s in cell parameters are only used when they are defined by crystal symmetry. An approximate (isotropic) treatment of cell e.s.d.'s is used for estimating e.s.d.'s involving l.s. planes.
Refinement. Refinement of *F*^2^ against ALL reflections. The weighted *R*-factor *wR* and goodness of fit *S* are based on *F*^2^, conventional *R*-factors *R* are based on *F*, with *F* set to zero for negative *F*^2^. The threshold expression of *F*^2^ > σ(*F*^2^) is used only for calculating *R*-factors(gt) *etc*. and is not relevant to the choice of reflections for refinement. *R*-factors based on *F*^2^ are statistically about twice as large as those based on *F*, and *R*- factors based on ALL data will be even larger.

### Fractional atomic coordinates and isotropic or equivalent isotropic displacement parameters (Å^2^)

**Table:**

	*x*	*y*	*z*	*U*_iso_*/*U*_eq_	
C1	0.2307 (2)	0.21698 (19)	0.03818 (12)	0.0464 (4)	
C2	0.2155 (3)	0.3435 (2)	0.09351 (15)	0.0675 (5)	
H2A	0.2195	0.4487	0.0448	0.101*	
H2B	0.3249	0.2973	0.1422	0.101*	
H2C	0.0922	0.3666	0.1295	0.101*	
C3	0.2508 (2)	−0.02273 (19)	0.01050 (12)	0.0450 (4)	
C4	0.2584 (3)	−0.2021 (2)	0.02881 (14)	0.0574 (4)	
H4A	0.2743	−0.2287	−0.0357	0.086*	
H4B	0.1369	−0.2134	0.0596	0.086*	
H4C	0.3691	−0.2805	0.0744	0.086*	
C5	0.2491 (2)	0.3310 (2)	−0.54342 (13)	0.0518 (4)	
C6	0.2507 (4)	0.2018 (3)	−0.59340 (17)	0.0790 (6)	
H6A	0.2633	0.0947	−0.5416	0.119*	
H6B	0.3612	0.1843	−0.6389	0.119*	
H6C	0.1286	0.2421	−0.6324	0.119*	
C7	0.2362 (2)	0.5735 (2)	−0.52294 (12)	0.0500 (4)	
C8	0.2197 (3)	0.7564 (2)	−0.54624 (15)	0.0691 (5)	
H8A	0.2259	0.7821	−0.4830	0.104*	
H8B	0.0951	0.8304	−0.5817	0.104*	
H8C	0.3274	0.7752	−0.5892	0.104*	
N1	0.2638 (2)	0.08533 (18)	−0.07827 (10)	0.0528 (4)	
N2	0.2508 (2)	0.23853 (17)	−0.06052 (10)	0.0533 (4)	
N3	0.22893 (18)	0.05496 (15)	0.08561 (9)	0.0433 (3)	
N4	0.2136 (3)	−0.02566 (19)	0.19138 (10)	0.0543 (4)	
H4D	0.100 (3)	0.047 (3)	0.2139 (15)	0.071 (6)*	
H4E	0.320 (3)	−0.030 (3)	0.2220 (15)	0.071 (6)*	
N5	0.2546 (2)	0.46293 (18)	−0.43183 (10)	0.0568 (4)	
N6	0.2637 (2)	0.30785 (18)	−0.44498 (11)	0.0579 (4)	
N7	0.2326 (2)	0.49581 (17)	−0.59485 (9)	0.0511 (4)	
N8	0.2096 (4)	0.5651 (3)	−0.70320 (12)	0.0790 (6)	
H8D	0.105 (4)	0.659 (4)	−0.710 (2)	0.109 (10)*	
H8E	0.325 (5)	0.589 (4)	−0.726 (2)	0.119 (10)*	
O1W	−0.1595 (3)	0.1723 (3)	0.26863 (18)	0.1022 (7)	
H1WA	−0.219 (5)	0.280 (4)	0.254 (2)	0.132 (12)*	
H1WB	−0.248 (5)	0.128 (4)	0.254 (2)	0.131 (11)*	
O2W	0.3524 (2)	0.47375 (18)	−0.23848 (12)	0.0706 (4)	
H2WA	0.308 (4)	0.412 (3)	−0.186 (2)	0.090 (7)*	
H2WB	0.308 (4)	0.466 (3)	−0.296 (2)	0.101 (8)*	
O3W	0.3962 (2)	0.02300 (16)	−0.26616 (11)	0.0610 (4)	
H3WA	0.341 (3)	0.055 (3)	−0.2121 (18)	0.079 (6)*	
H3WB	0.349 (4)	0.114 (3)	−0.321 (2)	0.093 (8)*	

### Atomic displacement parameters (Å^2^)

**Table:**

	*U*^11^	*U*^22^	*U*^33^	*U*^12^	*U*^13^	*U*^23^
C1	0.0471 (8)	0.0429 (8)	0.0438 (8)	−0.0139 (6)	−0.0002 (6)	−0.0069 (6)
C2	0.0849 (13)	0.0529 (10)	0.0645 (11)	−0.0241 (9)	−0.0015 (10)	−0.0161 (9)
C3	0.0393 (7)	0.0485 (8)	0.0455 (8)	−0.0143 (6)	0.0017 (6)	−0.0128 (7)
C4	0.0574 (10)	0.0543 (10)	0.0636 (11)	−0.0213 (8)	0.0020 (8)	−0.0198 (8)
C5	0.0514 (9)	0.0550 (9)	0.0478 (9)	−0.0184 (7)	0.0075 (7)	−0.0152 (7)
C6	0.0942 (15)	0.0765 (13)	0.0778 (14)	−0.0334 (11)	0.0145 (11)	−0.0378 (11)
C7	0.0513 (9)	0.0539 (9)	0.0444 (9)	−0.0212 (7)	0.0050 (7)	−0.0115 (7)
C8	0.0833 (13)	0.0610 (11)	0.0660 (12)	−0.0345 (10)	0.0060 (10)	−0.0129 (9)
N1	0.0571 (8)	0.0584 (8)	0.0431 (7)	−0.0227 (6)	0.0055 (6)	−0.0134 (6)
N2	0.0589 (8)	0.0512 (8)	0.0455 (8)	−0.0221 (6)	0.0029 (6)	−0.0053 (6)
N3	0.0445 (7)	0.0430 (7)	0.0374 (7)	−0.0144 (5)	0.0013 (5)	−0.0060 (5)
N4	0.0632 (9)	0.0543 (8)	0.0378 (7)	−0.0206 (7)	0.0038 (7)	−0.0034 (6)
N5	0.0697 (9)	0.0578 (8)	0.0427 (8)	−0.0249 (7)	0.0031 (6)	−0.0119 (6)
N6	0.0709 (9)	0.0516 (8)	0.0467 (8)	−0.0213 (7)	0.0030 (6)	−0.0086 (6)
N7	0.0565 (8)	0.0584 (8)	0.0368 (7)	−0.0242 (6)	0.0045 (6)	−0.0080 (6)
N8	0.1082 (16)	0.0910 (14)	0.0374 (8)	−0.0448 (13)	0.0018 (9)	−0.0062 (8)
O1W	0.0737 (10)	0.0831 (12)	0.170 (2)	−0.0312 (9)	0.0195 (10)	−0.0655 (13)
O2W	0.1040 (11)	0.0668 (9)	0.0514 (8)	−0.0482 (8)	−0.0005 (7)	−0.0090 (6)
O3W	0.0781 (9)	0.0496 (7)	0.0488 (7)	−0.0161 (6)	0.0067 (6)	−0.0143 (6)

### Geometric parameters (Å, °)

**Table:**

C1—N2	1.300 (2)	C7—N7	1.352 (2)
C1—N3	1.362 (2)	C7—C8	1.483 (2)
C1—C2	1.478 (2)	C8—H8A	0.9600
C2—H2A	0.9600	C8—H8B	0.9600
C2—H2B	0.9600	C8—H8C	0.9600
C2—H2C	0.9600	N1—N2	1.391 (2)
C3—N1	1.304 (2)	N3—N4	1.4091 (18)
C3—N3	1.355 (2)	N4—H4D	0.93 (2)
C3—C4	1.482 (2)	N4—H4E	0.88 (2)
C4—H4A	0.9600	N5—N6	1.389 (2)
C4—H4B	0.9600	N7—N8	1.411 (2)
C4—H4C	0.9600	N8—H8D	0.88 (3)
C5—N6	1.299 (2)	N8—H8E	0.93 (3)
C5—N7	1.354 (2)	O1W—H1WA	0.84 (3)
C5—C6	1.474 (3)	O1W—H1WB	0.92 (3)
C6—H6A	0.9600	O2W—H2WA	0.87 (3)
C6—H6B	0.9600	O2W—H2WB	0.90 (3)
C6—H6C	0.9600	O3W—H3WA	0.88 (2)
C7—N5	1.299 (2)	O3W—H3WB	0.89 (3)
			
N2—C1—N3	109.30 (14)	N5—C7—C8	126.38 (16)
N2—C1—C2	126.85 (15)	N7—C7—C8	124.62 (15)
N3—C1—C2	123.85 (15)	C7—C8—H8A	109.5
C1—C2—H2A	109.5	C7—C8—H8B	109.5
C1—C2—H2B	109.5	H8A—C8—H8B	109.5
H2A—C2—H2B	109.5	C7—C8—H8C	109.5
C1—C2—H2C	109.5	H8A—C8—H8C	109.5
H2A—C2—H2C	109.5	H8B—C8—H8C	109.5
H2B—C2—H2C	109.5	C3—N1—N2	107.78 (13)
N1—C3—N3	109.02 (14)	C1—N2—N1	107.36 (12)
N1—C3—C4	126.61 (15)	C3—N3—C1	106.55 (13)
N3—C3—C4	124.37 (14)	C3—N3—N4	124.23 (13)
C3—C4—H4A	109.5	C1—N3—N4	129.20 (13)
C3—C4—H4B	109.5	N3—N4—H4D	106.6 (12)
H4A—C4—H4B	109.5	N3—N4—H4E	105.8 (13)
C3—C4—H4C	109.5	H4D—N4—H4E	109.1 (17)
H4A—C4—H4C	109.5	C7—N5—N6	107.56 (14)
H4B—C4—H4C	109.5	C5—N6—N5	107.64 (13)
N6—C5—N7	108.89 (15)	C7—N7—C5	106.91 (14)
N6—C5—C6	126.70 (17)	C7—N7—N8	129.47 (15)
N7—C5—C6	124.41 (16)	C5—N7—N8	123.59 (15)
C5—C6—H6A	109.5	N7—N8—H8D	102.0 (18)
C5—C6—H6B	109.5	N7—N8—H8E	105.6 (17)
H6A—C6—H6B	109.5	H8D—N8—H8E	112 (3)
C5—C6—H6C	109.5	H1WA—O1W—H1WB	106 (3)
H6A—C6—H6C	109.5	H2WA—O2W—H2WB	107 (2)
H6B—C6—H6C	109.5	H3WA—O3W—H3WB	107 (2)
N5—C7—N7	109.00 (15)		

### Hydrogen-bond geometry (Å, °)

**Table:**

*D*—H···*A*	*D*—H	H···*A*	*D*···*A*	*D*—H···*A*
N4—H4D···O1W	0.93 (2)	2.00 (2)	2.924 (3)	170.9 (18)
N4—H4E···O3W^i^	0.88 (2)	2.21 (2)	3.078 (3)	168.3 (18)
N8—H8E···O2W^ii^	0.93 (3)	2.23 (3)	3.104 (3)	156 (2)
O1W—H1WA···O2W^iii^	0.84 (3)	1.95 (3)	2.793 (3)	173 (3)
O1W—H1WB···O3W^iv^	0.92 (3)	1.93 (3)	2.810 (2)	160 (3)
O2W—H2WA···N2	0.87 (3)	2.02 (3)	2.885 (2)	171 (2)
O2W—H2WB···N5	0.90 (3)	1.93 (3)	2.816 (2)	168 (2)
O3W—H3WA···N1	0.88 (2)	1.92 (2)	2.787 (2)	168 (2)
O3W—H3WB···N6	0.89 (3)	1.93 (3)	2.827 (2)	176 (2)

Symmetry codes: (i) −*x*+1, −*y*, −*z*; (ii) −*x*+1, −*y*+1, −*z*−1; (iii) −*x*, −*y*+1, −*z*; (iv) −*x*, −*y*, −*z*.
